# Gender effects of single nucleotide polymorphisms and miRNAs targeting *clock-genes* in metastatic colorectal cancer patients (mCRC)

**DOI:** 10.1038/srep34006

**Published:** 2016-09-26

**Authors:** Carlo Garufi, Elisa Giacomini, Angela Torsello, Isabella Sperduti, Elisa Melucci, Marcella Mottolese, Massimo Zeuli, Giuseppe Maria Ettorre, Teresa Ricciardi, Francesco Cognetti, Mauro Magnani, Annamaria Ruzzo

**Affiliations:** 1 Division of Medical Oncology, Spirito Santo Hospital Pescara, Italy; 2 Dept. of Biomolecular Sciences (DiSB) University of Urbino “Carlo Bo”, Urbino, Italy; 3 Division of Medical Oncology Azienda Ospedaliera San Giovanni Addolorata Hospital, Rome, Italy; 4 Biostatistic Unit, Regina Elena National Cancer Institute Rome, Italy; 5 Pathology Department Regina Elena National Cancer Institute Rome, Italy; 6 Division of Medical Oncology Regina Elena National Cancer Institute Rome, Italy; 7 General Surgery and Transplantation Unit San Camillo Hospital Rome, Italy

## Abstract

The circadian system is composed of a set of *clock-genes* including *PERIOD, CLOCK, BMAL1* and *CRY*. Disrupting this system promotes cancer development and progression. The expression levels of miR-206, miR-219, miR-192, miR-194 and miR-132 regulating *clock-genes* and three functional polymorphisms rs11133373 C/G, rs1801260 T/C, rs11133391 T/C in *CLOCK* sequence were associated with the survival of 83 mCRC patients (50 males and 33 females). Longer overall survival (OS) was observed in women compared to men, 50 versus 31 months. This difference was associated with rs11133373 C/C genotype (p = 0.01), rs1801260 T/C+C/C genotype (p = 0.06) and rs11133391 T/T genotype (p = 0.06). Moreover women expressing high levels (H) of miR-192 (p = 0.03), miR-206 (p = 0.003), miR-194 (p = 0.02) and miR-219 (p = 0.002) had a longer OS compared to men. In women longer OS was reinforced by the simultaneous presence of two or more H-miR, 58 months versus 15 months (p = 0.0008); in this group of women an OS of 87 months was reached with the additional presence of rs11133391T/T genotype (p = 0.02). In this study we identified a subgroup of female patients who seems to have a better prognosis. Personalized medicine should prospectively take into account both genetic and gender differences.

The circadian system is a coordinated network of biological clocks that drive 24 h changes in metabolism and proliferation with the suprachiasmatic nucleus (SCN) of the hypothalamus synchronizing circadian oscillations in most peripheral tissues, including the gastrointestinal tract. A molecular clock, based on circadian variation in a set of *clock-genes,* has been validated at a cellular level where two transcriptional factors Clock and Bmal1 (*CLOCK* and *BMAL1* gene) activate their targets, Per (*PERIOD1*, *2* and *3* gene) and Cry (*CRY1* and *2* gene)^1^,^2^. The Clock plays a central role in regulating the circadian rhythm. It is a transcription factor of the basic helix-loop-helix (bHLH) which contains DNA binding histone acetyltransferase activity. The clock forms a heterodimer with Bmal1 that binds E-box enhancer elements upstream of *PERIOD* and *CRY* and activates transcription of these genes. Subsequently Cry and Per interfere with Clock/Bmal1 activity repressing their own transcription by interacting in a negative feedback loop. Experimental data show that the disruption of the circadian rhythm is responsible for several metabolic-related diseases where increasing evidence has raised the possibility that the circadian rhythm genes may play an important role in human cancer development and progression^1^.

Large population-based studies have documented a relatively high degree of polymorphisms in the human *CLOCK* and have shown how several of them affect the subject’s phenotype^3^,^4^,^5^,^6^,^7^. Previous genetic association studies have proven that there are three functional single nucleotide polymorphisms in the *CLOCK* sequence linked to cancer and phenotype, these include: rs11133373 C/G and rs11133391 T/C, linked to prostate and breast cancer, and rs1801260 T/C, linked to several phenotypes, such as sleep disturbance, mood weight loss bipolar disorders, breast and CRC^2^,^4^,^6^,^7^,^8^,^9^. Karantanos *et al.*^7^, in a case-control study in 402 patients and 480 healthy controls showed that the 311T>C *CLOCK* gene polymorphism was associated with an increased risk of colorectal cancer development but with no difference in outcome. Alexander *et al.*^6^ confirmed an increased risk for adenoma in another case-control study when the *PERIOD3* gene length polymorphism was evaluated.

*Clock-genes* are regulated by different miRNAs and among these miR-192, miR-194, miR-206, miR-132 and miR-219 are associated with different types of cancer such as breast, lung and gastrointestinal cancer^10^,^11^,^12^,^13^,^14^,^15^,^16^. MiRNAs are small non coding RNA molecules that regulate gene expression by binding partially to complementary target sites within the 3′-untranslated regions (3′-UTRs) of select mRNAs. MiRNAs act as potent negative regulators of protein translation either by directing the mRNA for degradation or inhibiting its translation into the encoded protein. This level of regulation can affect virtually all aspects of the circadian system, from the core timing mechanism and input pathways that synchronize clocks to the environment and output pathways that control overt rhythmicity^11^.

Recently it has been reported that differences in survival are related to gender and to specific genetic profiling^17^,^18^.

In this study, for the first time, three functional polymorphisms in the *CLOCK* sequence, and five miRNAs regulating *CLOCK, BMAL1* and *PERIOD* in advanced colorectal cancer patients and their effect on survival were analyzed.

## Results

### Patients data

**Figure 1:**
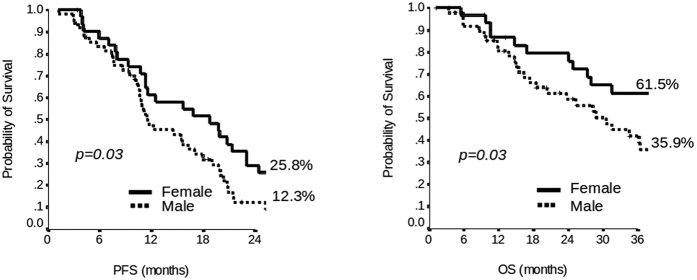
Kaplan-Meier plot of Progression Free Survival (PFS) at 2 years and Overall Survival (OS) at 3 years of patients according to sex.

Patient data are shown in Table 1. Most of the patients (78%) received first line triplet chemotherapy (fluorouracil plus oxaliplatin and irinotecan) ± monoclonal antibody, 82% were KRAS wild-type and 55% of them were resected for liver metastases after induction chemotherapy. Median progression free survival (PFS) was 14 months and median overall survival (OS) was 35 months. Women were observed to have better median PFS and OS than men, 19 months versus 12 months (2 years-PFS 25.8% vs 12.3%, p = 0.03) and 50 months versus 31 months (3 years-OS 61.5% vs 35.9%, p = 0.03), respectively (Fig. 1 and Table 1). Differences in prognostic factors between the female and male groups were also observed for age and chronomodulated therapy (p = 0.04), as reported in Table 1.

### Polymorphisms

Seventy six of the 83 the patients were genotyped for rs11133373 C/G, rs1801260 T/C and rs11133391 T/C polymorphisms. The genomic DNA of 7 cases was either consumed or degraded. No departures from Hardy-Weinberg equilibrium were observed and the frequencies did not show any differences when compared to the Italian population (TSI, Tuscany) (data not shown). No associations were observed in the entire population between polymorphisms and patient and tumour characteristics nor with clinical outcome, PFS and OS. Only performance status was linked to rs11133391 T/T genotype with a relative risk of 1.2 (95% CI 1–1.5; p = 0.04).

### rs11133373 C/G

**Figure 2:**
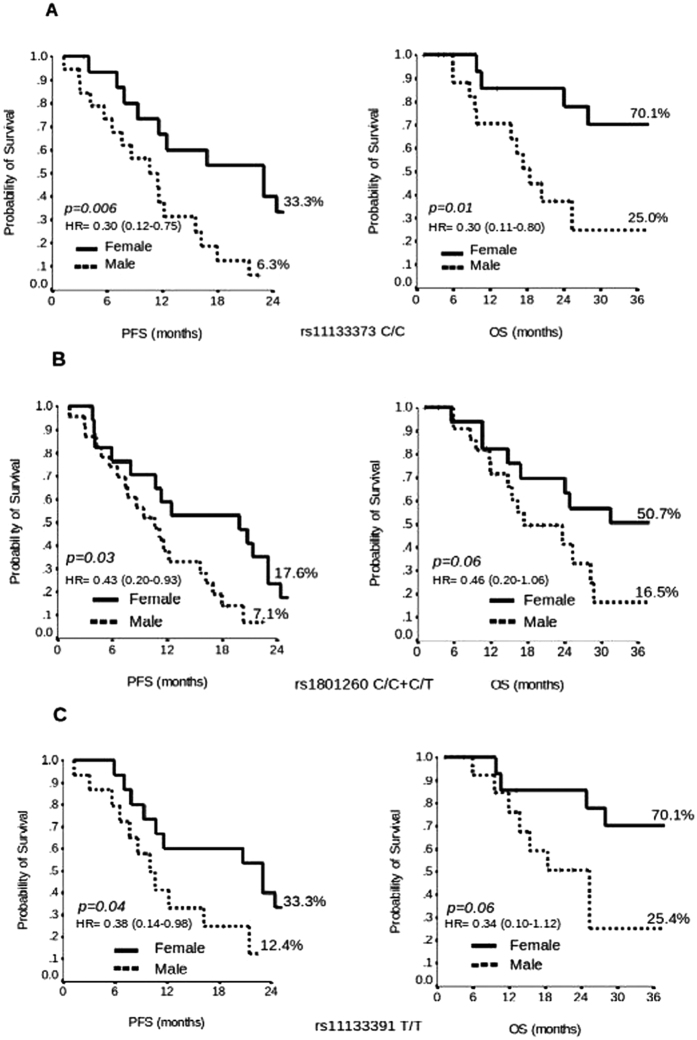
Kaplan-Meier plot of Progression Free Survival (PFS) at 2 years and Overall Survival (OS) at 3 years in Female versus Male with rs11133373 C/C genotype (**A**), rs1801260 T/C+C/C genotype (**B**) and rs11133391 T/T genotype (**C**).

When analysed by gender, results showed a better survival rate in women compared to men with rs11133373 C/C, median PFS of 23 months vs 11 months (2 years-PFS 33.3% vs 6.3%, p = 0.006) and median OS of 57 months vs 18 months (3 years-OS 70.1% vs 25.0%, p = 0.01), respectively (Fig. 2A). In women the C/C genotype was also associated with a longer PFS and OS compared to C/G+G/G genotype (23 months vs 16 months and 57 months vs 44 months, respectively) while the opposite effect happened in men (11 months vs 14 months and 18 months vs 32 months, respectively) (supplementary Table S1).

### rs1801260 T/C

Results showed a better survival rate in women compared to men with rs1801260 T/C+C/C genotype, median PFS of 20 months vs 11 months (2 years-PFS 17.6% vs 7.1%, p = 0.03) and median OS of 50 months vs 17 months (3 years-OS 50.7 vs 16.5%, p = 0.06), respectively (Fig. 2B).

### rs1133391 T/C

The rs11133391 T/T genotype was associated with longer median PFS of 23 months vs 10 months (2 years-PFS 33.3% vs 12.4%, p = 0.04) and median OS of 87 months vs 25 months (3 years-OS 70.1 vs 25.4% p = 0.06), respectively (Fig. 2C). We also found that the T/T genotype was associated with a longer OS compared to T/C+C/C genotype (87 months vs 44 months) (see supplementary Table S1). We did not find any differences between performance status and T/T genotype in patients divided by gender.

### miRNA expression levels

The expression levels of miR-192, miR-206, miR-194, miR-219 and miR-132 were successfully performed in 81 cases (49 males and 32 females). The expression levels of each miRNA were separated into High (H) and Low (L) categories and the association analysis between H and L of each miRNA and survival of patients were analyzed. No significant associations were found between miRNAs and patient data with the exception of hepatic resection: we found 12 patients with hepatic resection vs 29 without hepatic resection in the L-miR-194 group and 24 patients with hepatic resection vs 16 without hepatic resection in the H-miR-194 group reaching a relative risk of 1.77 (95% CI 1.15–2.71; p = 0.009).

### miRNAs expression levels and gender effect

When patients were divided by sex, women expressing H-miR-192, H-miR-206, H-miR-194 and H-miR-219 had a longer survival compared to men.

### miR-192

**Figure 3:**
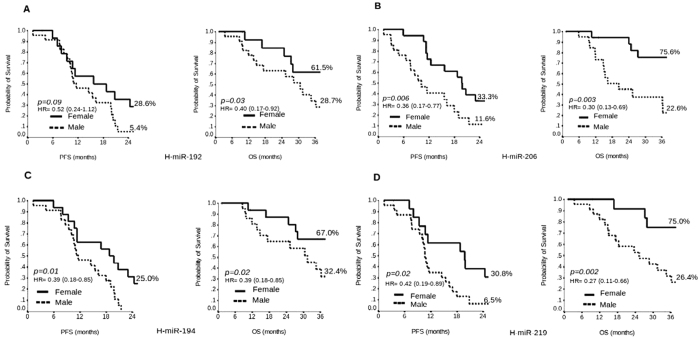
Kaplan-Meier plot of Progression Free Survival (PFS) at 2 years and Overall Survival (OS) at 3 years in Female versus Male with H-miR-192 (**A**), H-miR-206 (**B**), H-miR-194 (**C**) and H-miR-219 (**D**).

Women with H-miR-192 showed a median PFS of 16 months vs 12 months in men (2 years-PFS 28.6% vs 5.4%, p = 0.09) and a median OS of 51 months vs 31 months (3 years-OS 61.5% vs 28.7%, p = 0.03), respectively (Fig. 3A).

### miR-206

Women with H-miR-206 showed a median PFS of 20 months vs 10 months in men (2 years-PFS 33.3% vs 11.6%, p = 0.006) and median OS of 56 months vs 20 months (3 years-OS 75.6% vs 22.6%, p = 0.003), respectively (Fig. 3B). Women with H-miR-206 had a longer survival compared to those with L-miR-206 with an 11 month difference for PFS and 25 months for OS. In contrast men with H-miR-206 had a shorter survival compared to those with L-miR-206 with a 4 month difference for PFS and 12 months for OS (supplementary Table S2).

### miR-194

Women with H-miR-194 showed a median PFS of 19 months vs 12 months in men (2 years-PFS 25.0% vs 0.0%, p = 0.01) and median OS of 56 months vs 32 months (3 years-OS 67.0% vs 32.4%, p = 0.02), respectively (Fig. 3C).

### miR-219

Women with H-miR-219 showed a median PFS of 20 months vs 11 months (2 years-PFS 30.8% vs 6.5%, p = 0.02) and median OS of 58 months vs 25 months (3 years-OS 75.0% vs 26.4%, p = 0.002), respectively (Fig. 3D). Women with H-miR-219 had a longer survival compared to those with L-miR-219 with a 4 month difference for PFS and 8 months for OS. On the contrary men with H-miR-219 had a shorter survival compared to those with L-miR-219 with an 8 month difference for PFS and 6 months for OS (supplementary Table S2).

### miR-132

Women with L-miR-132 had a longer PFS and OS compared to those expressing H-miR-132 with a difference of 5 months and 13 months, respectively (supplementary Table S2).

**Figure 4:**
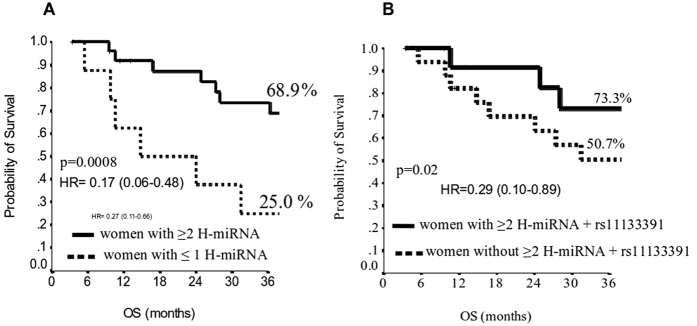
Kaplan-Meier plot of Overall Survival (OS) at 3 years, in women with ≥2 H*-*miR versus ≤1 H*-*miR (**A**) and in women with ≥2 H*-*miR + rs11133391 T/T genotype versus women without ≥2 H*-*miR + rs11133391 T/T (**B**).

Subsequently, an additional analysis was performed in women expressing two or more H*-*miR simultaneously versus none and we found that the first group had a median OS of 58 months versus 15 months (3 years-OS 68.9% vs 25.0%, p = 0.0008). This effect was further increased by the presence of rs11133391T/T genotype (2 years-OS 73.3% vs 50.7%, p = 0.02) with a median OS of 87 months vs 44 months, as shown in Fig. 4 (supplementary Table S3).

## Discussion

This study highlights that functional rs11133373 C/G, rs1801260 T/C, rs11133391 T/C *CLOCK* polymorphisms as well as expression levels of miR-192, miR-206, miR-194 and miR-219 related to *clock-genes*, are differently associated with survival in women and men with mCRC, suggesting that the circadian system plays a role in this gender-effect result. Moreover this study indicates that these genetic variants could be used as sex-specific prognostic biomarkers because an opposite expression effect in women and in men related to survival was found.

Treatment of advanced colorectal cancer has profoundly evolved over the last twenty years from mono chemotherapy with 5-fluorouracil modulated by folinic acid to double or triple combination chemotherapy plus a monoclonal antibody. Moreover the selection of RAS mutation has identified a subset of patients who cannot benefit from anti-EGFR antibodies such as cetuximab or panitumumab. These changes, together with the use of resection of liver metastases, was the reason for the survival shift from 12 months to more than 30 months^19^,^20^ as in this series with a median PFS of 14 months and median OS of 35 months. With reference to drugs delivery in cancer patients according to circadian rhythms, three trials compared conventional versus time-dependent administration of 5-fluorouracil-folinic acid and oxaliplatin. In the last of these trials improved survival was found in men treated with chronomodulated infusion and in women treated by conventional flat infusion^21^. When analyzed together, a meta-analysis confirmed a significant three-month median survival improvement in men versus women with chronoinfusion and the contrary in female patients receiving flat infusion suggesting a gender effect in the modulation of time-delivered treatment schedule^22^.

On the contrary, opposite results were found in these 83 advanced colorectal cancer patients. Most of them were administered a three drug combination ± cetuximab, female patients were significantly younger than men and were mostly treated by chronomodulated infusion. In this population female patients had significantly better median PFS (19 versus 12 months) and OS (50 versus 31 months) than men. Thus a possible explanation for relying on the different signatures in *clock-genes* regulation according to gender could be plausible.

Sexual dimorphism has been observed in most human diseases^23^,^24^,^25^. Both hormonal and genetic differences between men and women can lead to differences in gene expression patterns that can influence disease risk and course even in cancer. Although CRC is more frequent in men, prognosis seems to be better in women. Majek *et al.*^26^ showed in a German population of 164,996 patients an age-adjusted 5-year relative survival higher in women (64.5% vs 61.9%, p < 0.0001). This was then confirmed at the multivariate analysis in subjects under 65 years of age with an excess attributable risk of death lower than 14%. In this group, women have fewer adenomas but roughly equivalent to the number of colorectal cancers found in men. Simon *et al.*^27^ reported that women randomized to receive estrogen/progesterone in the Women’s Health Initiative had a lower rate of CRC but a greater number of positive lymph nodes than women in the placebo group. Female patients treated in the adjuvant setting benefit more than men after resection of colon cancer^26^,^27^,^28^. Gender related differences in survival were advocated for urothelial and kidney cancer^29^. Stage and gender were found to be prognostic factors for survival in lung cancer^30^. In a series of 11,774 melanoma cases a significant female advantage was observed for melanoma specific survival. They were at a lower risk of progression and maintained a significant survival advantage after first progression and lymph node metastases^31^.

Functional polymorphisms of EGFR are inversely related to gender specific OS in mCRC: the EGFR (CA)_n_ repeat polymorphism was associated with OS in female patients, with females carrying fewer than (CA)_20_ repeats having better OS and was associated to increased EGFR expression, while in males the opposite was observed^18^. Also estrogen receptor ß (Erß) polymorphisms have been shown to be relevant and gender related in advanced colorectal cancer^32^. Press *et al.*^18^ showed that female patients with less than (CA)_22_ repeat alleles had short OS compared to female patients that had in homozygous more than (CA)_22_ repeat alleles, while the opposite difference in survival was found in men. Finally a link between EGFR signaling and the circadian system has been shown. Lauriola *et al.*^33^ showed that in animals glucocorticoids inhibit signaling downstream of EGFR through a circadian control mechanism: EGFR signals are suppressed by glucocorticoids during the active phase (night-time in rodents) while EGFR signals are enhanced during the resting phase. It was also demonstrated that KRAS overexpression can disrupt the circadian clock in metastatic cell lines^34^. We used the miRDB data base (mirdb.org/miRDB) for predicting the potential of miRNA-mRNA interaction also in the EGFR pathway. We found that miR-192, miR-206 and miR-194 have binding sites on EGFR mRNA; miR-206, miR-132, miR-194 and miR-219 on KRAS mRNA; miR-192 on ESR2 (Erß) mRNA; and miR-219 on EGF mRNA. Thus our hypothesis is that the biological framework of colorectal cancer in women seems to be characterized by Erß signaling, EGFR activation and circadian clock control, all interacting mechanisms, involved in tumour growth.

This study has several limitations. It is a retrospective study with a limited number of patients, so it can only be used to generate a hypothesis. Whereas the main strength of the study is that all the patients were treated by the same clinical group and follow-up was sufficiently long. Prospective, cooperative studies are needed to explore the mechanisms by which *CLOCK* polymorphisms and miRNA expression levels related to *clock-genes* exert their effect on EGFR or on other pathways. In the era of precision medicine the goal of treating men and women differently with advanced colorectal cancer is a promising challenge.

## Methods

### Patients

The cohort of patients was retrospective and consisted of 83 patients diagnosed with mCRC treated in one single center. Patients were required to have a histologically confirmed diagnosis of advanced CRC and available Formalin-Fixed, Paraffin-Embedded tissue (FFPE) samples of the primary tumor and peripheral blood samples. All experiments were performed in accordance with relevant guidelines and regulations. The study was approved by the Ethics Commitee of the Regina Elena National Cancer Institute in Rome and signed informed consents were collected.

The primary end-point of the study was the evaluation of survival outcome according to the SNPs and miRNAs expression levels. Additional analyses were addressed to compare miRNAs and SNPs according to clinical features.

The genotype analysis of rs11133373 C/G, rs1801260 T/C and rs11133391 T/C polymorphisms was performed on genome DNA (gDNA) extracted from peripheral blood cells in patients. The miRNA expression level analysis of miR-192, miR-206, miR-132, miR-194 and miR-219 was performed on miRNAs extracted from FFPE samples.

See under “supplementary Material and Methods” the Table S4 for nucleic acids extraction (gDNA and miRNA) and PCR conditions, for the miRNAs expression level analysis see Figure S1(A–E).

### Statistical Method

rs11133373 C/G, rs1801260 T/C and rs11133391 T/C were checked for any deviations from the Hardy-Weinberg equilibrium. The frequencies of each SNP in the cohort of patients were compared with the general Italian population (TSI, http://www.ensembl.org/info/genome/variation/index.html). According to the frequency of the minor allele and the low frequency of the homozygous variant genotype, we decided to collapse the heterozygous and minor allele homozygous in all the analyses performed. The expression levels of miR-192, miR-206, miR-132, miR-194 and miR-219, were expressed as values obtained from the ΔCt equation of each miRNA to test with respect to RNU-6-2 used as reference gene (ΔCt = Ct_target_ − Ct_reference_). Median ΔCt values were employed for splitting miRNA expression levels into two groups, High expression level (H) and Low expression level (L).

The Chi-Square or Fisher Exact tests were used to estimate all associations between categorical variables. Quantitative variables were compared using the non-parametric Mann–Whitney test. The Odds Ratio (OR) and the 95% confidence intervals (95% CI) were estimated for each variable using the logistic univariate regression model.

Overall survival (OS) and Progression-free Survival (PFS) were calculated by the Kaplan-Meier product-limit method. PFS was calculated as the time from the start date of the first line therapy until date of progression or date of last follow-up evaluation. OS survival was calculated as the time from the start date of the first line therapy until to the date of death or last contact. Log-rank test was used to assess the differences between subgroups.

All reported P-values were based on two-sided tests, and a *P*-value of less than or equal to 0.05 was considered statistical significant.

SPSS software was used for all statistical evaluations (SPSS version 21.0, SPSS Inc., Chicago, Illinois, USA).

## Additional Information

**How to cite this article**: Garufi, C. *et al.* Gender effects of single nucleotide polymorphisms and miRNAs targeting *clock-genes* in metastatic colorectal cancer patients (mCRC). *Sci. Rep.* **6**, 34006; doi: 10.1038/srep34006 (2016).

## Supplementary Material

Supplementary Information

## Figures and Tables

**Table 1:** Patient data.

	No	%	Male	%	Female	%	p-value***
	83		50		33		
Median Age (years-range)	59 (30–85)	—	59 (44–85)	—	57 (30–73)	—	0.21
< = 59/59>	16/67	19/81	6/44	12/88	10/23	30/70	**0.04**
Performance Status: 0 vs 1/2	65/18	78/22	38/12	76/24	27/6	82/18	0.53
Menopause No/Yes	—	—	—	—	10/23	30/70	—
Primary tumor resected: Yes/No	76/7	92/8	44/6	88/12	32/1	97/3	0.23
Site metastases: liver/other sites	48/35	58/42	30/20	60/40	18/15	55/45	0.62
Metastases: synchronous/metachronous	75/8	90/10	43/7	86/14	32/1	97/3	0.14
Resection of liver metastases: Yes/No	36/47	43/56	18/32	36/64	18/15	55/45	0.10
KRAS mutated: No/Yes	68/15	82/18	42/8	84/16	26/7	80/21	0.55
BRAF mutated: No/Yes	63/2	99/3	36/1	97/3	27/1	96/4	0.99
BRAF missing	18	22	13	26	5	15	0.29
First line Therapy							
Triplet* combination	39	48	24	48	15	46	0.12
Triplet* + cetuximab	25	30	11	22	14	42	
Douplet** + cetuximab	12	15	9	18	3	9	
Douplet** ± bevacizumab	5	7	6	12	1	3	
Chronomodulated infusion: Yes/No	57/26	69/31	30/20	60/40	27/6	82/18	**0.04**
Response to first line therapy							
Complete response	2	2	2	4	0	0	0.64
Partial response	55	66	31	62	24	73	
Stable disease	16	19	10	20	6	18	
Progressive disease	8	10	6	12	2	6	
Not assessable	2	2	1	2	1	3	
Median follow-up (months)	25 (range 1–132)	—	20 (range 1–121)	—	41 (range 3–132)	—	**0.05**
Median OS (months)	35 (CI 95% 21.5–47.5)	—	31 (CI 95% 22.0–39.1)	—	50 (CI 95% 35.0–64.0)	—	**0.03**
Median PFS (months)	14 (CI 95% 10–18.9)	—	12 (CI 95% 7.6–15.5)	—	19 (CI 95% 10.7–26.8)	—	**0.03**

Triplets*: 5-fluorouracil, folinic acid, oxaliplatin or irinotecan.

Douplets**: 5-fluorouracil, folinic acid + oxaliplatin or irinotecan.

Log-rank test***.
