# Supplementary material for: Gender effects of single nucleotide polymorphisms and miRNAs targeting clock-genes in metastatic colorectal cancer patients (mCRC)
Source: Sci Rep. 2016 Sep 26;6:34006. doi: 10.1038/srep34006 (PMC5036027; doi:10.1038/srep34006)
Supplement: Supplementary Information [file srep34006-s1.pdf]

TITLE: Gender effects of single nucleotide polymorphisms and miRNAs targeting *clock-genes* in metastatic colorectal cancer patients (mCRC)

[1]Carlo Garufi*§, [2]Elisa Giacomini, [3]Angela Torsello, [4]Isabella Sperduti, [5]Elisa Melucci, [5]Marcella Mottolese, [6]Massimo Zeuli, [7]Giuseppe Maria Ettorre, [2]Teresa Ricciardi, [6]Francesco Cognetti, [2]Mauro Magnani and [2]Annamaria Ruzzo*§

*Carlo Garufi and Annamaria Ruzzo contributed equally to the study

[1]Division of Medical Oncology, Spirito Santo Hospital Pescara, Italy; [2] Dept. of Biomolecular Sciences (DiSB) University of Urbino "Carlo Bo", Urbino, Italy;. [3.]Division of Medical Oncology Azienda Ospedaliera San Giovanni Addolorata Hospital, Rome , Italy; [4] Biostatistic Unit, Regina Elena National Cancer Institute Rome, Italy; [5] Pathology Department Regina Elena National Cancer Institute Rome Italy; [6]Division of Medical Oncology Regina Elena National Cancer Institute Rome, Italy; [7] General Surgery and Transplantation Unit San Camillo Hospital Rome Italy.

SUPPLEMENTARY

Table S1. Gender-related survival for rs11133373 C/G, rs1801260 T/C and rs11133391 T/C polymorphisms

			PFS			OS		
	N° patients		Months Median (95%CI)		p-value*	Months Median (95%CI)		p-value*
	Males	Females	Males	Females		Males	Females	
	45	31	12(8-16)	19(11-27)	**0.03**	31(22-39)	50(35-64)	**0.03**
**Polymorphisms**								
**rs11133373**								
C/C	19	16	11(6-17)	23(10-36)	**0.006**	18(14-23)	57(31-84)	**0.01**
C/G+G/G	26	15	14(9-20)	16(6-25)	0.41	32(23-40)	44(16-73)	0.35
**rs1801260**								
T/T	22	13	14(8-21)	17(8-25)	0.17	35(27-42)	57(46-68)	0.07
T/C+C/C	23	18	11(7-14)	20(7-33)	**0.03**	17(5-29)	50(21-78)	0.06
**rs11133391**								
T/T	15	15	10(7-13)	23(9-37)	**0.04**	25(14-37)	87(28-147)	0.06
T/C+C/C	30	16	12(7-16)	16(9-23)	0.29	29(19-38)	44(14-75)	0.31
Log-rank test*								

Table S2. Association analysis of PFS and OS between H-miRNAs and L-miRNA and gender

			PFS			OS		
			Months Median (95%CI)		p-value*	Months Median (95%CI)		p-value*
	Males	Females	Males	Females		Males	Females	
N° of patients	49	32	12(8-16)	19(11-27)	**0.03**	31(22-39)	50(35-64)	**0.03**
miR-192								
L	23	17	11(9-14)	20(10-30)	0.23	24(8-39)	50(19-80)	0.28
H	24	15	12(6-17)	16(2-29)	0.09	31(23-38)	51(14-88)	**0.03**
miR-206								
L	26	14	14(9-20)	9(0-20)	0.9	32(23-40)	31(7-56)	0.67
H	21	18	10(6-14)	20(17-22)	**0.006**	20(8-33)	56(46-66)	**0.003**
miR-132								
L	23	17	12(5-19)	21(8-34)	0.14	31(14-47)	57(15-99)	0.06
H	24	15	11(9-13)	16(4-27)	0.19	25(16-35)	44(5-84)	0.11
miR-194								
L	24	16	11(9-14)	16(5-26)	0.37	24(10-37)	50(16-83)	0.22
H	23	16	12(13-24)	19(13-24)	**0.01**	32(20-43)	56(35-77)	**0.02**
miR-219								
L	23	18	19(9-29)	16(7-25)	0.6	31(12-49)	50(19-80)	0.81
H	24	24	11(10-12)	20(10-30)	**0.02**	25(11-39)	58(55-61)	**0.002**
*Log-rank test								

Table S3. Overall survival in the subgroup of women with two or more H-miR ± rs11133391 T/T genotype

	Overall Survival		
	% at 3 years	Median 95% CI	p-value*
Female patients	61.5	50 (35-64)	
women ≥ 2 H-miR	68.9	58 (45-69)	**0.0008**
women ≤ 1 H-miR	25	15 (0-34)	
women ≥ 2 H-miR + rs11133391 T/T	73.3	87 (46-129)	**0.02**
women wothout ≥ 2 H-miR + rs11133391 T/T	50.7	44 (12-77)	
Log-rank test*			

SUPPLEMENTARY METHODS

DNA extraction and genotyping

gDNA was extracted by means of QIAmp DNA Blood kit (Qiagen, Hilden, Germany) according to the manufacturer's protocol. gDNA purity and concentration was measured by Nanodrop ND-1000 spectrophotometer (Nanodrop Technologies, Rockland, DE). Pyrosequencing technique has been used to detect rs11133373 C/G, rs1801260 T/C and rs11133391 T/C polymorphisms.

Primers for PCR amplification and pyrosequencing were chosen by pyromark software (Biotage) and are listed in supplementary Table S4.

All PCR reactions were carried out for 40 cycles in a total volume of 25µl containing 10ng of gDNA, 0.2 µM of each primer (forward and reverse), 12.5 µl PCR Master Mix (Diatheva, Fano, Italy) and 0.625 U HotStarTaq polymerase (Diatheva). Reaction parameters were 95°C for 10 min followed by 40 cycles of 95°C for 30 s, 59°C for 20 s and 72°C for 30 min. A final extension at 72°C was carried out for 3 min. Successful and specific amplification of the region of interest was verified by visualizing 5 µl of the PCR product on a 2% agarose gel electrophoresis. The Pyrosequencing

technique was performed on a PSQ 96MA instrument (Biotage) using PyroGold reagents (Qiagen) following the protocol suggested by the manufacturers and the determination of rs11133373 C/G, rs1801260 T/C and rs11133391 T/C polymorphisms were made by using PyroMark™ ID program (Qiagen).

miRNAs extraction and Quantitative Real-Time Polymerase Chain Reaction (q-RT-PCR)

Three to five 10-µm sections from FFPE specimens were obtained from the primary tumor. Representative areas from FFPE tumor blocks were evaluated by pathologists. Before cutting sections for miRNAs isolation, one slide was prepared for hematoxylin and eosin staining to select only representative samples with almost complete tumor infiltration. All assays were performed by investigators who were blinded to the clinical data of the sample cohort.

Total cellular RNA was isolated from human FFPE specimens using the miRNeasy FFPE Kit (Qiagen) according to the manufacturer's instructions. The extracted RNA was quantified and its purity was evaluated by the NanoDrop 1000 spectrophotometer (Nanodrop Technologies) and 250 ng of total RNA was reverse transcribed using the miScript II RT Kit (Qiagen) according to the manufacturer's instructions. Conditions for the reverse transcription (RT) reaction were as follows: 37°C for 60 minutes and 95°C for 5 minute. Obtained cDNA was diluted 1:11 and used as template in the q-RT-PCR, mixed with QuantiTect SYBR Green PCR Master Mix and miScript Universal Primer (Qiagen) and loaded into each well of a Custom miScript miRNA PCR Arrays made to include the set of miScript Primer Assay (Qiagen)of miR-192, miR-206, miR-132, miR-194 and miR-219 according to the manufacturer's instructions. The analysis was performed on an ABI-PRISM 7500 Real Time PCR System (Applied Biosystems, Foster City, CA, USA) using the manufacturer’s recommended program. Data were quantified using the SDS 2.1 software and normalized using RNU6-2 as endogenous control. The cycle threshold (Ct) value, which was calculated relatively to the endogenous control (ΔCt), was used to evaluate the relative changes in miRNA expression levels. The expression levels of each miRNA was expressed as value obtained from the Δct equation with respect to RNU-6-2 reference gene ($\Delta ct = Ct_{target} - Ct_{reference}$).

Table S4. Primers for PCR amplification and pyrosequencing. See supplementary Methods for PCR conditions

**ID**	**Primer sequence**	**Amplicon length**
rs11133373	F-biot-5'-GTTGTAAATCCCATTTCTGCAAATA-3'<br>R-5'-CCAAGAACCAATTTCAGGTTTTACT-3'<br>S-5'-TTACTCTTGCCTCCGT-3'	107bp
rs1801260	F-5'-CCAGCAGGAGGTGATCATAGG-3'<br>R-biot-5'-CAGGCACCTAAAACACTGTCAG-3'<br>S-5'-GGTGATCATAGGGGCA-3'	56bp
rs11133391	F-biot-5'-CAAATGAAATAACCCATTACCTTTAGA-3'<br>R-5'-AAGATTGATTAGCCATGAGTTGATAATT-3'<br>S-5'-TGGAAACTGGGTGAAG-3'	163bp
Legend:<br>F=forward; R=reverse; S=pyrosequencing primer; biot=biotinylated primer; bp= base pairs		

Figure S1 – Histograms (A, B, C, D, E) show the results of miRNAs expression analysis. Value are expressed in 2^-ΔCt*10000 to make more clear the result. For details about the expression analysis please see “miRNAs extraction and Quantitative Real-Time Polymerase Chain Reaction (q-RT-PCR)” section.

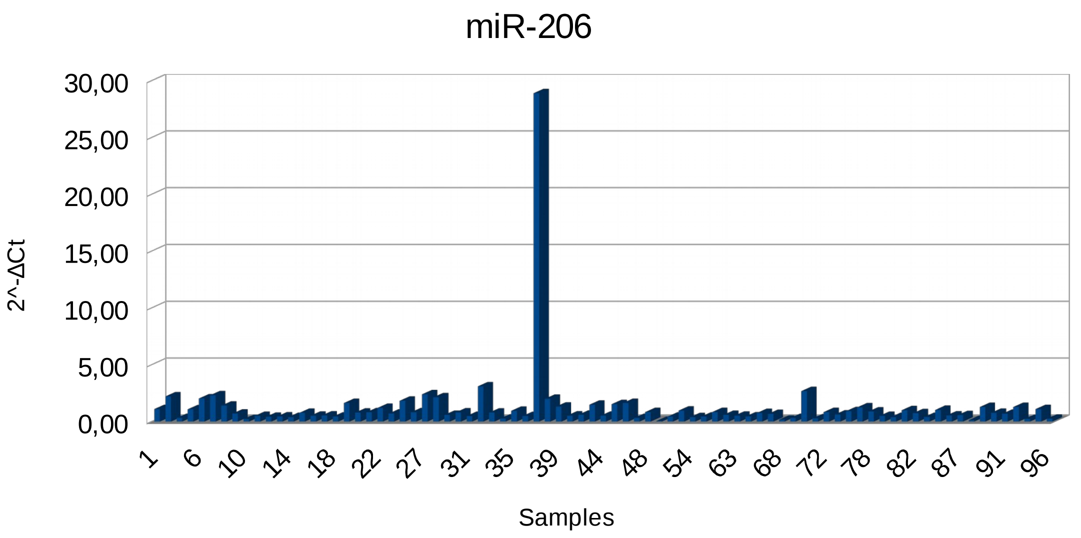

A

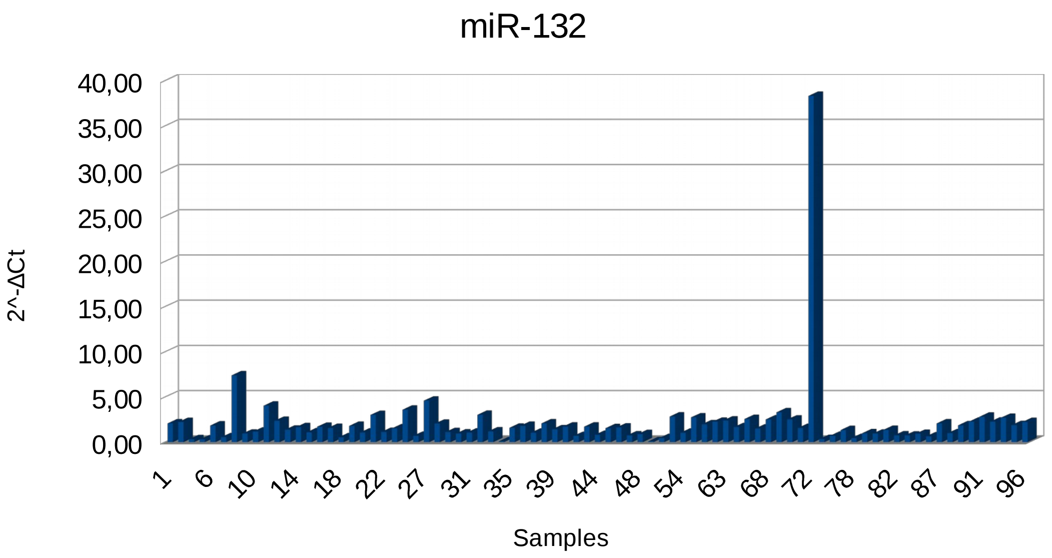

B

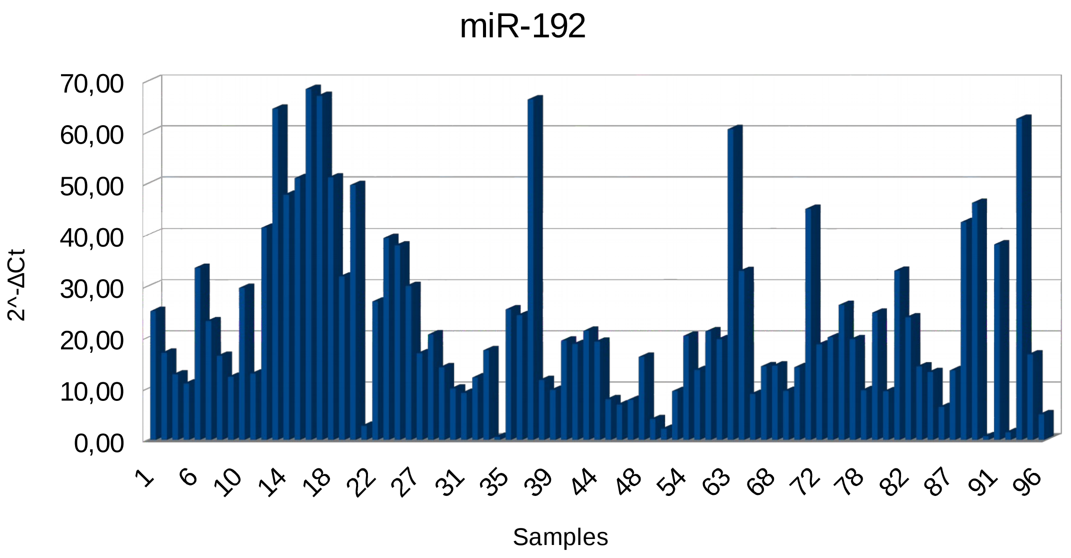
miR-192
70,00
60,00
50,00
40,00
30,00
20,00
10,00
0,00
2^-ΔCt
1
6
10
14
18
22
27
31
35
39
44
48
54
63
68
72
78
82
87
91
96
Samples

C

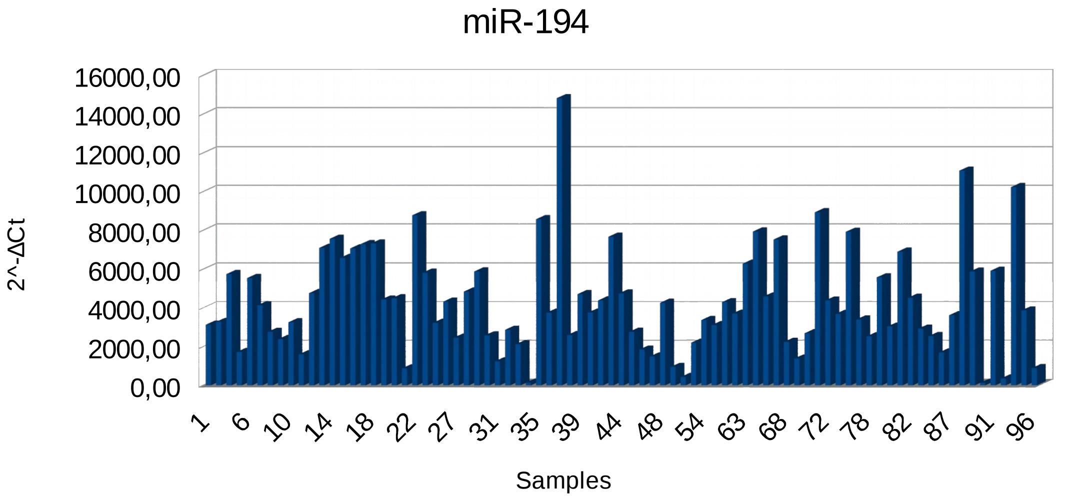
miR-194
16000,00
14000,00
12000,00
10000,00
8000,00
6000,00
4000,00
2000,00
0,00
2^-ΔCt
1
6
10
14
18
22
27
31
35
39
44
48
54
63
68
72
78
82
87
91
96
Samples

D

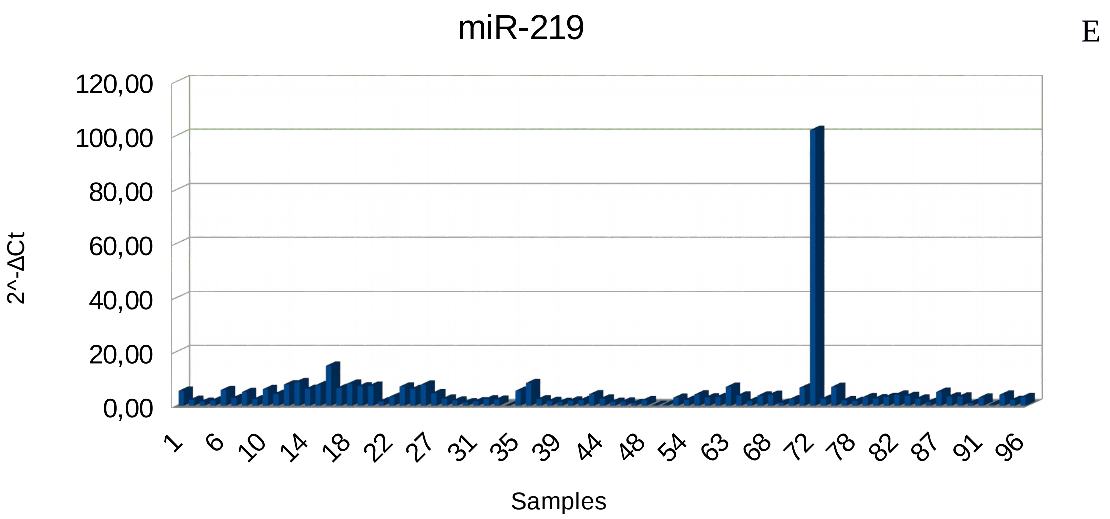
miR-219
E
120,00
100,00
80,00
60,00
40,00
20,00
0,00
2^-ΔCt
1
6
10
14
18
22
27
31
35
39
44
48
54
63
68
72
78
82
87
91
96
Samples
